# Tolerance to Herbicides and Resistance to Antibacterial Drugs of Bacterial Isolates From the Guarani Aquifer System (Brazil)

**DOI:** 10.1111/1462-2920.70115

**Published:** 2025-06-19

**Authors:** Carolina S. O. Silva, Sílvia D. Oliveira, Audrey M. Proenca, Eduarda V. Abati, Letícia Marconatto, Cássio S. Moura, Renata Medina‐Silva

**Affiliations:** ^1^ Geobiology Group, Laboratory of Immunology and Microbiology, School of Health and Life Sciences Pontifical Catholic University of Rio Grande do Sul, PUCRS Porto Alegre Brazil; ^2^ Institute of Petroleum and Natural Resources Pontifical Catholic University of Rio Grande do Sul, PUCRS Porto Alegre Brazil; ^3^ Institute of Biology, Freie Universität Berlin Berlin Germany

**Keywords:** 2,4‐D, antibiotic resistance, bacterial isolates, environmental health, glyphosate, pesticides

## Abstract

The contamination of water bodies by pesticides and antibiotics is a concerning environmental problem on a global scale. We investigated the impact of commonly used herbicides and antibiotics on bacterial isolates from the Guarani Aquifer System (GAS), the second largest aquifer in the world, in agriculture‐intensive regions in southern Brazil. A total of 23 isolates were exposed to 2,4‐D and glyphosate‐based herbicides. Among these, 19 were tolerant (some presenting increased survival) to at least one herbicide. The collection site had a significant effect on isolates' maximum survival (MS), and a strong cross‐tolerance between the two chemicals was detected, whereas seven out of 13 isolates (genera *Bacillus*, *Lysinibacillu*s, *Pseudomonas* or *Enterococcus*) were resistant to at least one antibiotic. Isolates with the highest index of antibiotic resistance showed high MS values to herbicides, suggesting cross‐resistance. We present the first characterisation of herbicide and antibiotic susceptibility of bacteria isolated from a deep aquifer. Herbicide tolerance was high and common, showing correlation with antibiotic resistance. The results suggest herbicides may impact microbial communities in aquifers, particularly concerning GAS—which spans four countries in South America—highlighting the importance of studying environmental microbes as potential remediators of contaminants, in line with the One Health principle.

## Graphical abstract

Sites in Southern Brazil (RS state) where water samples from the Guarani Aquifer System were collected and from which bacteria were isolated and further tested for herbicides tolerance and antimicrobial resistance.

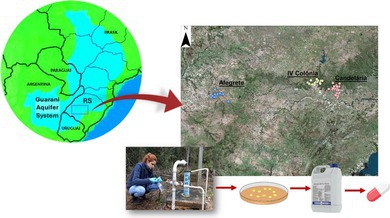

## 1 Introduction

Brazil has been the world's leading pesticide consumer since 2008 (Pignati et al. 2017). The use of pesticides in rural activities impacts the environment, and it is possible to find evidence of contamination by these substances in samples from water reservoirs and other aquatic environments (Chiarello et al. 2017). Among the most used pesticides in Brazil (ANVISA 2018), the herbicides glyphosate and 2,4‐dichlorophenoxyacetic acid (2,4‐D) are broadly applied to a wide variety of crops in the Brazilian southern region (Talha‐Mar Soluções Ambientais 2010). However, their indiscriminate use might have unintended consequences on environmental microbial populations.

Glyphosate is an aminophosphonate analogous to the natural amino acid glycine. It competitively inhibits the enzyme 5‐enolpyruvylshikimate‐3‐phosphate (EPSPS), transferring a carboxyvinyl group from phosphoenolpyruvate (PEP) to shikimate‐3‐phosphate (S3P) (Schönbrunn et al. 2001). First sold commercially in 1974, it has become the most common and intensively used herbicide in the world, applied in the control of weeds in a variety of agricultural and non‐agricultural environments. In Brazil, over 30 formulations of glyphosate are registered and marketed (Brazilian Ministry of Agriculture, Livestock and Supply; https://www.gov.br/agricultura/pt‐br). Moreover, varying levels of glyphosate sensitivity have been reported for the microbial EPSPS enzyme. This fact not only suggests that this compound could impact microorganisms, but evidence also indicates the toxicity of this herbicide to microbial species (Rainio et al. 2021; Bonnet et al. 2007; Pizarro et al. 2016). Similarly, 2,4‐D is a synthetic auxin derived from phenoxyacetic acid and registered for commercial use since the 1940s (Burns and Swaen 2012). Due to its commercial formulation in the form of salts, amine and ester, 2,4‐D is rapidly metabolised and classified as biodegradable, not persisting on the water surface (Wilson et al. 1997). However, a study reported that 2,4‐D residues can promote changes in the structure of microbial communities in soil (Aguiar et al. 2020), indicating that it should not be considered an inert and harmless compound to ecosystems. Moreover, it can also be toxic to non‐target organisms, like bacterial species (de Castro Marcato et al. 2017).

Beyond the direct impact to the composition of soil microbiomes, herbicides can change the response of microorganisms to antibiotics (Brigitta et al. 2015). For instance, simultaneous exposure of *Escherichia coli* and *Salmonella enterica* populations to commercial herbicides and antibiotics promoted changes in susceptibility to drugs of several classes (Brigitta et al. 2015). Moreover, exposure to commercial pesticide formulations can lead to the evolution of antibiotic resistance, with the response of microbial populations varying according to the combination of bacterial strain, antibiotics and pesticides used during the experiment (Kurenbach et al. 2018). An increase in antibiotic survival can also result from adaptive resistance (Fernández and Hancock 2012), which can be triggered by antimicrobials and/or other environmental stressors (Palmer and Kishony 2013).

Naturally, the evolution of cross‐resistance to antimicrobial agents is a concern that extends far beyond laboratory settings. Antimicrobial contamination in water bodies, due to urban wastewater and agriculture, is a growing problem worldwide (Kurenbach et al. 2018; Novo and Manaia 2010). Residues of antimicrobials and other pollutants in the environment, even at low concentrations, exert a selective pressure for resistant bacteria (Ohore et al. 2019). Antibiotic‐resistant bacteria and their resistance genes spread through aquatic environments, which can introduce genetic changes into bacterial populations in natural ecosystems. Thus, in such systems, environmental bacteria may act as an unlimited source of genetic elements carrying resistance genes that can reach pathogenic organisms, leading to an increased risk for human health (Resende et al. 2020). For example, research on marine bacteria found that over 90% of their sea‐sediment isolates were resistant to more than one antibiotic, and 20% to at least five different antimicrobials (Yang et al. 2013). Xenobiotic exposure in aquatic environments may thus lead to cross‐resistance to various antimicrobial substances in wild microbial communities (Rangasamy et al. 2018).

As herbicides have long been introduced into agricultural environments and spread through water bodies, endemic bacterial populations resistant to antimicrobials may have already emerged. Several parallel studies report that the use of herbicides leads to the accumulation of these compounds in the environment, impacting microbial communities in different ecosystems and potentially selecting herbicide‐tolerant and antimicrobial‐resistant populations (de Castro Marcato et al. 2017; Yang et al. 2013; Mohanty and Jena 2019; Pileggi et al. 2020; Qiu et al. 2022). As aquifers are connected to surface water and sediment, these chemicals, as well as tolerant/resistant bacterial strains, can reach groundwater mainly during flood events, inducing significant changes in microbial communities' composition (Fillinger et al. 2021). In this context, studies that evaluate the emergence of antibiotic resistance in groundwater systems, like aquifers, are increasingly frequent (Zainab et al. 2020; Andrade et al. 2020; Anand et al. 2021; Junaid et al. 2022). Nevertheless, we lack a characterisation of cross‐resistance to pesticides and antimicrobials in microbial strains from these aquatic systems.

The Guarani Aquifer System (GAS) is one of the most important hydrostatic systems in the southern portion of South America. The Paraná River Basin, which houses the GAS, is the most important hydrogeological province in Brazil. It has about 45% of the underground water reserves of the entire national territory and, due to its ability to store and release large amounts of water, it is a source of water for family consumption, industry and agriculture (Sindico et al. 2018). In the southern Brazilian state, Rio Grande do Sul (RS), the GAS spans along a large rural area with intense agricultural and livestock production activities that have extensively employed the use of herbicides for at least five decades (Pignati et al. 2017; ANVISA 2018; Talha‐Mar Soluções Ambientais 2010; Sindico et al. 2018). Chemical analysis performed on water samples from the GAS sites in south Brazil indicated the presence of the pesticide 2,4‐D (Soares 2019), among other chemical compounds, such as heavy metals in high concentrations (Moreno et al. 2021). These results indicate that this underground water storage system may be suffering anthropic impact, most probably from surface infiltration of these pollutants from surface water and sediment (Fillinger et al. 2021; Moreno et al. 2021). In this context, this study is the first to analyse bacterial isolates from water samples of the GAS along three regions of RS (Brazil), investigating and characterising their susceptibility to herbicides and antibiotics.

## 2 Methods

### 2.1 Origin of Bacterial Isolates

Bacterial isolates were previously obtained from water collected during a sampling campaign in 2018 in the state of Rio Grande do Sul (Brazil), in which more than 30 artesian wells connected to the GAS were sampled for geophysical, chemical (Soares 2019; Moreno et al. 2021) and/or microbiological analysis (Figure S1). For this study, water samples were aseptically collected from nine different wells from three regions—Candelária (two wells: Candelária and Várzea do Botucaraí), Alegrete (three wells: Caverá 1, Caverá 2 and Capivari) and Quarta Colônia (four wells: Gruta Sítio Alto, Gruta dos Mellos, Caemborá and Riacho Felis)—(Figure S1, Table 1). The sampling sites (wells) identification, depth, region and coordinates (in Universal Transverse Mercator, UTM) (Soares 2019; Moreno et al. 2021), as well as the number of bacterial isolates obtained from each one, are indicated in Table 1. Among these, the site MEL was the only one in which 2,4‐D was previously detected (at 0.004 mg/L) (Soares 2019), whereas no data on glyphosate detection was reported for any of these sites so far. The isolates were then preserved in 30% glycerol and stored at −80°C.

**TABLE 1:** Sampling sites of the Guarani Aquifer System water samples from which the bacterial isolates were previously obtained.

Region	City	Sampling sites				No. of isolates
		Name (abbreviation)	Well depth (meters)	UTM X	UTM Y	
Candelária	Candelária	Candelária (C)	91	326,940	6,716,545	4
		Várzea do Botucaraí (VB)	60	328,378	6,690,715	3
Alegrete	Alegrete	Caverá 1 (CAV1)	104	641,697	6,679,200	1
		Caverá 2 (CAV2)	112	639,680	6,679,013	3
		Capivari (CAP)	123	601,401	6,696,673	6
Quarta Colônia	Faxinal do Soturno	Gruta Sítio Alto (GRU)	40	253,331	6,731,263	1
		Gruta dos Mellos (MEL)	100	257,279	6,728,510	2
	Nova Palma	Caemborá (CAE)	140	277,021	6,737,934	1
		Riacho Felis (RF)	60	275,426	6,740,767	2

*Note:* The names, well depth, coordinates (in UTM) and number of bacterial isolates of each site, as well as their region and city in Rio Grande do Sul state (Brazil), are indicated.

*Source:* Soares (2019) and Moreno et al. (2021).

For the present study a total of 23 isolates were selected to be tested. This group included only isolates that belong to bacterial genera that are predicted by CLSI or EUCAST for antimicrobial testing. Additionally, isolates with similar growth rates were selected. Isolates were all recovered in BHI (Brain Heart Infusion) broth, at 28°C, for 24–48 h. These cultures were then plated on BHI agar and incubated at 28°C for 24–48 h for colony isolation. The colonies were analysed under light microscopy (1000×) after Gram staining to confirm the isolate purity and morphology. For all herbicide treatments and antimicrobial susceptibility analyses, *Pseudomonas aeruginosa* ATCC 27853, *Bacillus cereus* ATCC 33019 and *Enterococcus faecalis* ATCC 29212 were used as reference strains.

### 2.2 Taxonomic Identification of Bacterial Isolates

For taxonomic identification of bacterial isolates, DNA was extracted using the QIAamp DNA Stool Mini Kit (Qiagen). The complete sequence of the 16S rRNA gene was amplified by PCR using the following primers: 9 forward (5′ AGA GTT TGA TCC TGG CTC AG 3′) and 1542 reverse (5′ AGA AAG GAG GTG ATC CAG CC 3′) (Edwards et al. 1989). Amplification was performed in a 50 μL mixture, consisting of 1.5 mM MgCl_2_, 0.2 μM of each primer, 0.2 mM of each dNTP, 1 U Platinum *Taq* DNA polymerase, 1× PCR reaction buffer and approximately 10 ng of genomic DNA. PCR conditions used were the following: an initial activation at 94°C for 2 min, and 25 cycles of 45 s at 94°C, 45 s at 55°C and 60 s at 72°C, followed by an extension at 72°C for 3 min. The reaction products were purified using the Wizard SV Gel and PCR Clean‐Up System (Promega) and sequenced by the capillary method by ACTGene Análises Moleculares (Nova Alvorada, RS, Brazil). The forward and reverse sequencing reads were assembled and trimmed into single contigs using the software DNA Sequence Assembler version 5.15.0 (*Phred quality score cutoff of* < 20). Contigs were then aligned against the NCBI database through the Basic Local Alignment Search Tool (BLAST). Similar and reference sequences were downloaded from the NCBI database to perform a phylogenetic analysis. Sequences were further aligned using the ClustalW tool incorporated in MEGA X (Kumar et al. 2018). Phylogenetic analyses were performed using the Phylogeny Tool on MEGA X. The phylogenetic trees were constructed using the maximum likelihood method considering a threshold of 95% nucleotide identity and the Tamura‐Nei model (Tamura and Nei 1993). Statistical significance was measured by 1500 bootstrap replications. The 16S rRNA sequences from isolates that were taxonomically identified were deposited in the NCBI database: C9 (OM949960), C14 (OM949967), CAE1 (OM949968), CAP2 (OM949990), CAV19 (OM952179), CAV211 (OM952208), GRU33 (OM952259), MEL33 (OM952437), VB1 (OM952920) and VB4 (OM952921).

### 2.3 Herbicide Treatments

Bacteria were grown in BHI broth (at 25°C for 24–48 h) and the optical density of cultures was measured at 600 nm using a spectrophotometer. The cultures were washed with 0.9% saline to remove the growth medium. Bacterial suspensions were prepared in 0.9% saline at a cell density equivalent to the 0.5 McFarland standard. The isolates were exposed to a concentration gradient of glyphosate (4, 6 and 8 μg/mL) and 2,4‐D (1.2, 1.5 and 1.8 μg/mL) separately in 96‐well plates (Bellinaso et al. 2003). The concentration ranges used for each pesticide exceeded by at least a factor of 10 the respective Maximum Permitted Values (MPVs) established by the Brazilian National Council for the Environment (CONAMA) Resolution No. 396 (2008) for all groundwater use categories (CONAMA 2008). The control group was exposed to sterile 0.9% saline solution. These tests were performed without nutrients, aiming to verify bacterial tolerance to both 2,4‐D and glyphosate, which is the ability to maintain survival in the presence of these chemicals. This strategy simultaneously allows the possibility to verify the occurrence of bacteria that can grow using these herbicides as the sole nutrients source, which can be an indication of the ability to degrade such chemicals.

Four different exposure times were chosen for dilution and seeding of aliquots: 5, 20, 30 and 45 h. These exposure times were chosen based on previous pilot growth curve experiments conducted by the research group, which indicated these time points were the most relevant for observing differences in survival among our isolates (unpublished data). After exposure, each culture was diluted at 10^−3^, 10^−4^ and 10^−5^, drop‐plated (10 μL) in triplicate on BHI agar, and cultured at 28°C for 24 h for subsequent estimation of colony‐forming units per ml (CFU/mL). Data were expressed as relative changes to CFU/mL counts (herbicide exposure/control), in log2 scale. Changes in growth were considered biologically meaningful when the means ± relative errors (standard deviation/mean) had no overlap with the 95% confidence intervals (CIs) around zero change in growth (log2 CFU fold change = 0). These CIs were defined as

$$95\%\mathrm{CI}=\bar{x}\pm t\frac{\sigma }{\sqrt{n}}\;\mathrm{df}=n-1$$

where *x̄* represents the mean, *σ* the standard deviation and *n* the sample size. *t* values were obtained from the t distribution for the corresponding degrees of freedom (df): for our sample sizes, there is a 95% probability that a sample would fall between −4.30 and 4.30, which on a log2 scale results in an interval between −2.105 and 2.105 (represented in the shaded area of plots in Figures 2, 3 and S1). Values beyond this interval were considered a sign of either growth promotion or sensitivity, whereas values within these limits suggested no meaningful change in growth and survival due to herbicide exposure.

There are no standard criteria for considering bacteria sensitive, tolerant, or resistant to herbicides, since different studies use distinct parameters to classify bacterial species or isolates regarding this profile. In our experiments, we considered isolates presenting non‐significant changes in CFU/mL counts as ‘tolerant’, and those with a significant decrease in these values as ‘sensitive’ to herbicides. Isolates that increased survival under herbicides' exposure were also considered tolerant and additionally ‘herbicide‐promoted’, which can be used as equivalent to the classification as ‘highly tolerant’, in previous studies (Mohanty and Jena 2019; Qiu et al. 2022; Curutiu et al. 2017; Olchanheski et al. 2014). The maximum survival (MS) was determined as the maximum CFU fold change relative to unexposed controls. For each isolate and herbicide combination, we recorded MS along with the exposure time at which it occurred, and the corresponding herbicide concentration. These parameters were used for further analyses (see Section 2.5). The MS proved to be a useful parameter, consistently detectable across all isolate's survival assays, facilitating accurate comparisons of herbicide tolerance.

### 2.4 Antimicrobial Susceptibility Tests

Isolates properly identified as belonging to the genera *Bacillus*, *Lysinibacillus*, *Pseudomonas*, or *Enterococcus* were firstly evaluated for their antibiotic susceptibility by the disk‐diffusion method, according to EUCAST standard methods. These genera were selected since they represent bacterial taxa currently reported as occurring in aquatic environments, including the GAS (Vilas‐Boas et al. 2024). In this context, *Staphylococcus* and *Leuconostoc* were not included for these tests. These genera are not usually detected in such habitats and mostly include potential pathogenic species. The test was performed employing the antimicrobials amikacin, aztreonam, ceftazidime, clindamycin, erythromycin, cefepime, gentamicin, imipenem, linezolid, levofloxacin, meropenem, tobramycin and/or vancomycin, according to EUCAST indication for each bacterial genus. The results of the disk‐diffusion test were interpreted using the breakpoints of inhibition zones' diameter available for *Bacillus*, *Pseudomonas* and *Enterococcus*, following EUCAST guidelines.

For those that presented resistance to at least two antimicrobial drugs, we calculated the multiple antibiotic resistance (MAR) index (Krumperman 1983). MAR index, when applied to a single isolate, is defined as *a/b*, where *a* represents the number of antibiotics to which the isolate was resistant, and *b* represents the number of antibiotics to which the isolate was exposed (Krumperman 1983).

Minimum inhibitory concentrations (MICs) were also determined for representatives of different classes of antibiotics in isolates that indicated resistance to them in the disk‐diffusion tests. This test indicates the minimum concentration of the antimicrobial drug that is able to inhibit bacterial growth, based on the turbidity of bacterial cultures tested in 96‐well plates. *Pseudomonas* isolates were tested for ceftazidime (cephalosporin), tobramycin (aminoglycoside) and aztreonam (monobactam). The *Bacillaceae* family was tested for meropenem (carbapenem), erythromycin (macrolide), clindamycin (lincosamide) and vancomycin (glycopeptide). MICs were determined by the microdilution method according to EUCAST standards. The breakpoints for resistance adopted were those established by the EUCAST guidelines for these antibiotics, considering the bacterial genera submitted to the test.

### 2.5 Statistical Analysis

Statistical analyses were performed using R (version 3.6.1) (R Core Team 2019). MS values obtained for each isolate were log2‐transformed prior to visualisation and analysis, and the data was tested for normality (Shapiro–Wilk test) and homogeneity of variance (Levene's test) to verify test assumptions. To compare MS values of isolates as a function of the sampling region (i.e., city), a *one‐way* ANOVA and the Tukey HSD (Honestly Significant Difference) post hoc test were applied. The correlation between MS values of both herbicides was evaluated through a linear regression and Pearson's correlation test.

A Principal Component Analysis (PCA) was performed using the R package ‘vegan’ (Mandal et al. 2020). Each data vector was centred around 0 and scaled (divided by the standard deviation) prior to analysis. Five dimensions were investigated, consisting of log_2_‐transformed MS values from glyphosate and 2,4‐D treatments, and the diameters of inhibition zones from disk diffusion tests of three broad‐spectrum antibiotics (imipenem, ciprofloxacin and levofloxacin). The diameters of inhibition zones were centred at zero using the resistance threshold value for each antibiotic/isolate pair, such that positive values indicated susceptibility and negative values indicated resistance. This transformation was also applied prior to heatmap visualisation. The PCA was performed to determine the components that contribute the most to the variance within and among groups of isolates, and to provide a 2D representation of this data. A Permutational Multivariate Analysis of Variance (PERMANOVA) was performed on the 5D data to determine significant patterns in the grouping of isolates regarding both antibiotic and herbicide susceptibility, using the Euclidean method to evaluate distances between groups. *p* values less than 0.05 were considered indicative of significance.

## 3 Results

### 3.1 Taxonomic Identification of Isolates

**FIGURE 1:**
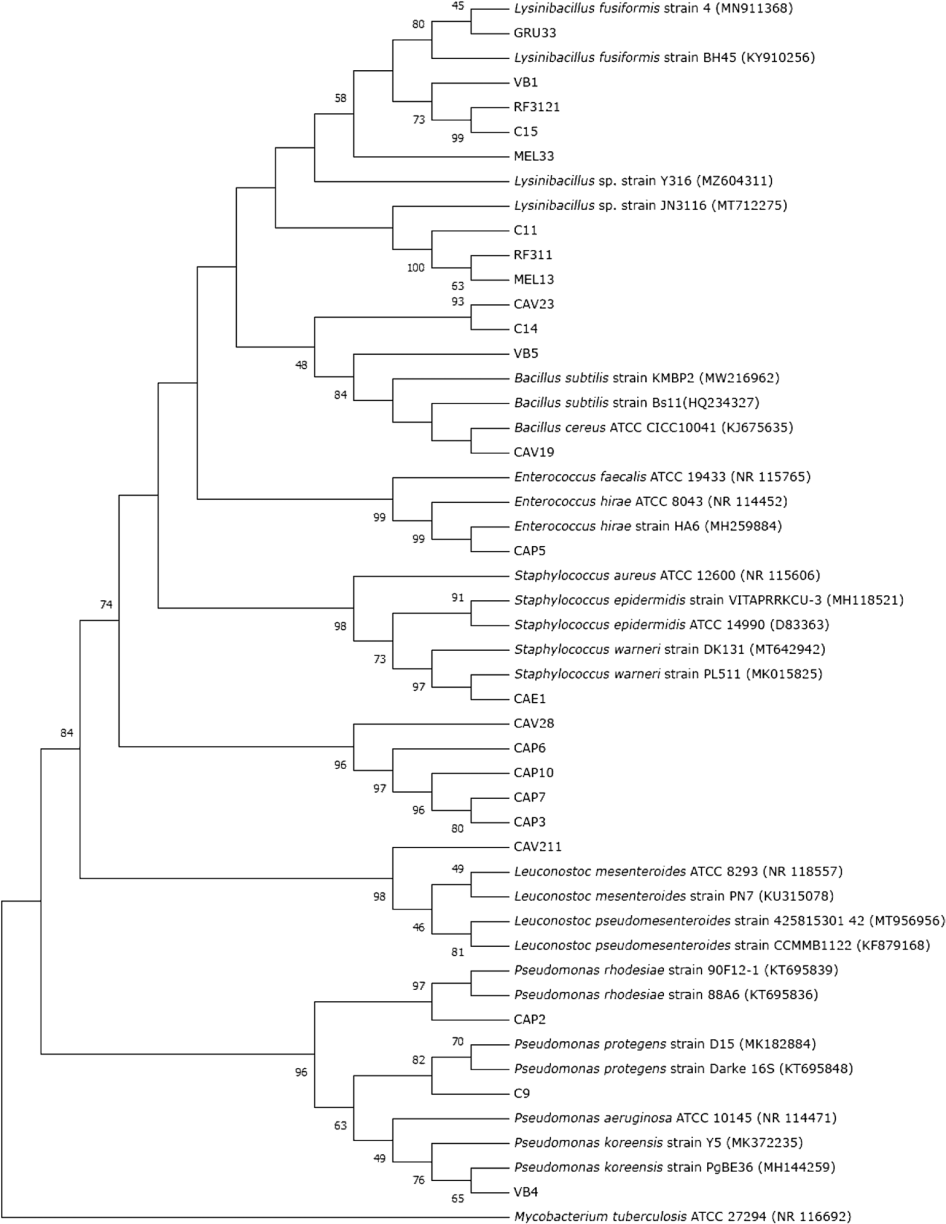
Taxonomic identification of 23 bacterial isolates obtained from the Guarany Aquifer System. Phylogenetic analyses were performed with reference sequences obtained from the Nucleotide BLAST database. Phylogenetic trees were constructed using the maximum likelihood method and Tamura‐Nei model based on 16S rRNA gene sequences. Bootstrap percentages based on 1500 replications are shown at branch points (values below 45 were cut off).

We performed the taxonomic identification of 23 isolates from the GAS, based on the sequencing of the 16S rRNA gene and on phylogenetic analyses considering a threshold of 95% nucleotide identity (Figure 1). Among the isolates, 9 presented a phylogenetic relationship with genera from the *Bacillaceae* family. From these, 5 isolates (C15, GRU33, MEL33, RF3121 and VB1) showed similarity with sequences of *Lysinibacillus* reference strains, most belonging to the *L. fusiformis* species. Three other isolates (C11, MEL13 and RF311) also grouped within the *Lysinibacillus* clade, but with a low bootstrap value that could not support their taxonomic identification. Moreover, 4 isolates (C14, CAV19, CAV23 and VB5) formed a separate clade with *B. cereus* and *B. subtilis* reference sequences. Apart from the *Bacillaceae* isolates, 3 bacteria grouped within a clade with reference sequences of the *Pseudomonas* genus, each being phylogenetically close to a distinct species: *P. rhodesiae* (CAP2), *P. protegens* (C9) and *P. koreensis* (VB4). Also, the isolate CAP5 grouped with *E. faecalis* and *E. hirae* strains, CAV211 within a *Leuconostoc mesenteroides* and *L. pseudomesenteroides* clade, and CAE1 with *Staphylococcus* strains (more related to *S. warneri* ), all supported by moderate to high bootstrap values.

Moreover, Figure 1 illustrates 5 isolates (CAP3, CAP6, CAP7, CAP10 and CAV28) that showed no close phylogenetic relationship with the reference sequences employed in this analysis, and together formed a distinct clade, in which they shared high similarity. Most of these bacteria were isolated from the same collection site (CAP, see Table 1), which may in part explain their phylogenetic proximity.

### 3.2 Herbicide Treatments

**FIGURE 2:**
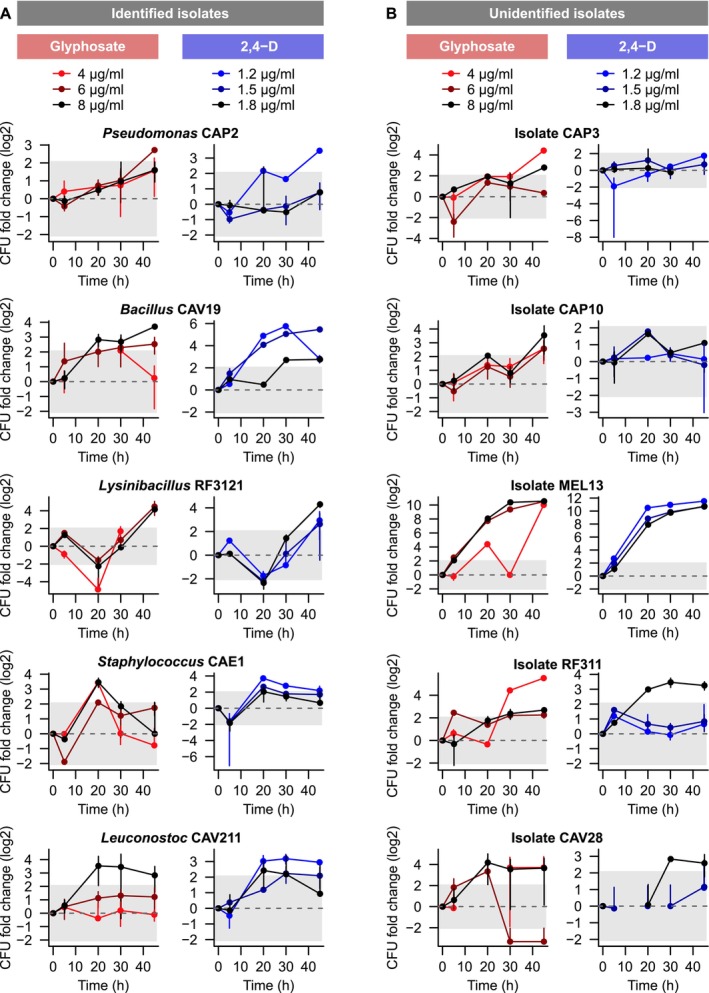
Environmental isolates showing growth promotion under herbicides' exposure. Ten isolates showed a positive change in CFU/ml counts, relative to control cultures unexposed to glyphosate and 2,4‐D herbicides. In each panel, the dashed line represents no change in growth due to herbicide exposure (log2 CFU fold change = 0), while the shaded area represents the 95% confidence intervals around which no biologically meaningful growth improvement or sensitivity is observed (see Methods for details). (A) Five identified and (B) five unidentified isolates had their survival increased by at least one herbicide concentration along the treatments. Error bars represent the relative error (standard deviation/mean) of each treatment. CFU fold changes were considered biologically meaningful when means ± relative error reached beyond the 95% confidence intervals.

**FIGURE 3:**
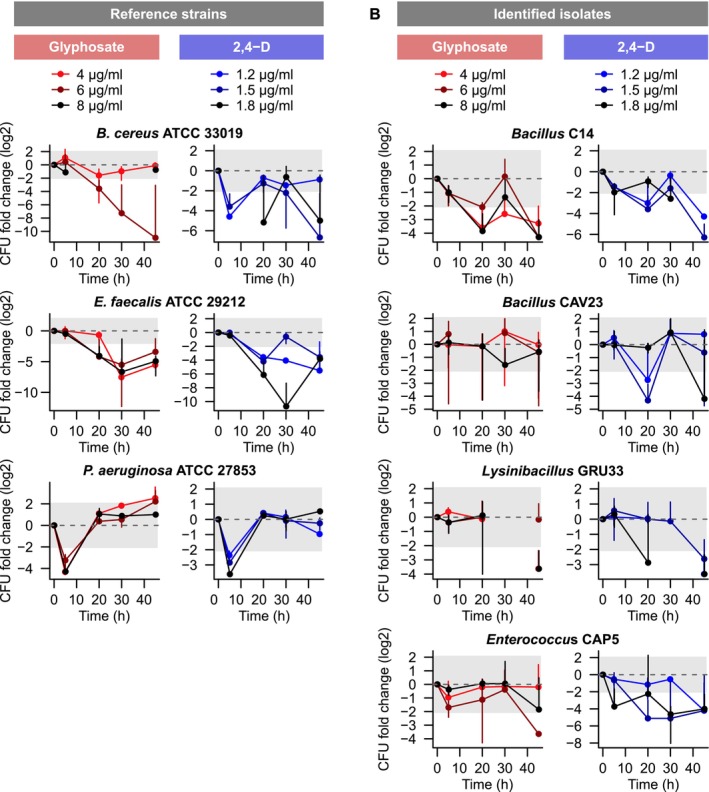
Strains showing sensitivity to herbicides' exposure. (A) Reference strains and (B) four identified isolates suffered a negative effect of pesticide treatment on growth, relative to unexposed controls. As in Figure 2, the dashed line represents no change in growth (log2 CFU fold change = 0), while the shaded area represents the 95% confidence intervals around which no biologically meaningful change is observed. Error bars = mean ± relative error.

Heterogeneous responses among the isolates were observed, with Figure 2 showing herbicide‐tolerant isolates and Figure 3 depicting sensitive strains. In each plot, the dashed line represents no impact of herbicide exposure on growth, and the shaded area represents the 95% CI beyond which tolerance or sensitivity were considered biologically meaningful. With rare exceptions, such as the progressive growth improvement of MEL13 in all treatments (Figure 2) and the gradual decrease in *B. cereus* survival under 6 μg/mL (Figure 3), a clear response to treatment duration was rarely observed. Nevertheless, different from the reference strains, which showed significantly decreased survival compared to control, the majority of environmental isolates were tolerant or had their growth significantly promoted under most concentrations of both herbicides. It is relevant to highlight that the herbicides were the sole source of nutrients, carbon and energy for the isolates during the exposure period.

Of the 15 isolates that were identified taxonomically, 6 exhibited herbicide tolerance, showing no growth changes due to either treatment (Figure S1A). More importantly, five identified isolates displayed increased growth in the presence of herbicides (Figure 2A). *Pseudomonas* CAP2 had a slight growth promotion due to glyphosate and 2,4‐D treatments, which increased after 45 h of exposure to 6 μg/mL glyphosate and 1.2 μg/mL 2,4‐D. *Bacillus* CAV19 exhibited a considerable growth improvement in most concentrations of both herbicides, reaching its MS value when treated with 1.2 μg/mL 2,4‐D for 30 h. *Lysinibacillus* RF3121 presented a peculiar behaviour, with a tendency to show sensitivity after 20 h, followed by increased survival after 45 h for most concentrations of both herbicides. This might indicate an adaptation period of this isolate to the stressful herbicide encounter, after which growth improvement can be observed. *Staphylococcus* CAE1 showed the highest growth levels after 20 h of treatment for both herbicides, whereas *Leuconostoc* CAV211 showed efficient growth for all treatments, even under the highest concentration of glyphosate.

The remaining four isolates out of the 15 taxonomically identified ones showed sensitivity to herbicide treatments, though not as pronounced as that of reference strains (Figure 3). *Bacillus* C14 had increased sensitivity over time, with lowest growth observed after 45 h of treatment (Figure 3B). *Bacillus* CAV23 presented a similar behaviour, showing high sensitivity to 2,4‐D in at least two time points. *Lysinibacillus* GRU33 was sensitive to both herbicides at 45 h, and *Enterococcus* CAP5 showed a tendency to be sensitive to both herbicides, with a significant decrease in survival after 45 h of glyphosate exposure and a dosage‐dependent growth decrease for 2,4‐D treatments over time. The reference strains *B. cereus* ATCC 33019 and *E. faecalis* ATCC 29212 were sensitive to both herbicides. *P. aeruginosa* ATCC 27853 presented a distinct behaviour, with considerable sensitivity after 5 h of exposure but reaching some level of tolerance after a longer exposure to either herbicide (Figure 3A). In total, 6 *Bacillaceae* isolates showed either growth improvement or tolerance due to the treatment, as did all *Pseudomonas* other than the reference strain.

Regarding the eight unidentified isolates (3 unsupported *Lysinibacillus* and five isolates that formed a distinct clade), all showed either tolerance (Figure S2) or growth promotion (Figure 2B) at least under one herbicide concentration. Two of the unsupported *Lysinibacillus* (MEL13 and RF311) were among the herbicide‐promoted isolates, with MEL13 showing the largest CFU/ml fold change among all tested strains (Figure 2B). This growth improvement was present for all concentrations of both herbicides and increased with the duration of each treatment. Unidentified isolates CAP3 and CAP10 also showed growth promotion, whereas CAV28 exhibited a mixed pattern. This isolate had high survival levels under the highest concentration of each herbicide under at least one time point but showed sensitivity to intermediate glyphosate concentrations after 30 h of exposure. More importantly, no unidentified isolates showed sensitivity to pesticides.

**FIGURE 4:**
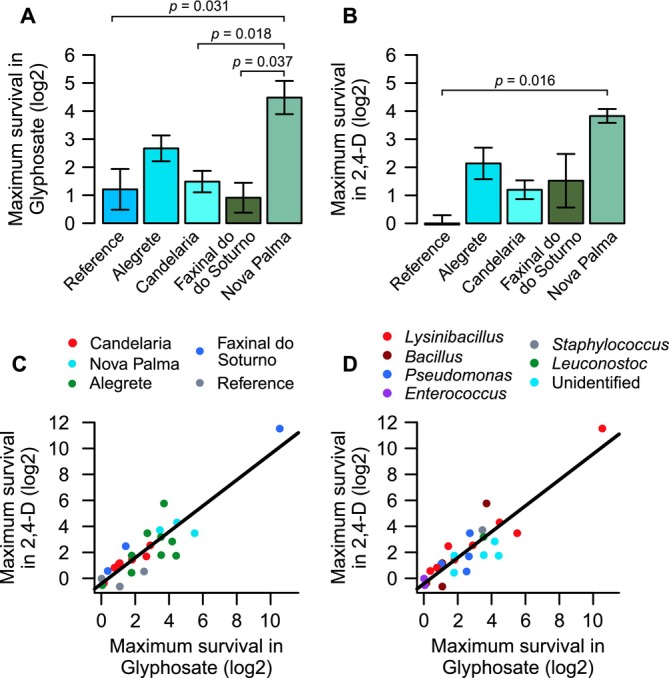
Maximum survival (MS) trends across isolates and collection regions. MS in the presence of (A) glyphosate and (B) 2,4‐D was higher for isolates obtained from Nova Palma, with the reference strains showing the lowest MS after 2,4‐D exposure. Error bars = mean ± SEM. (C and D) Across the 23 isolates and 3 reference strains, MS values of both herbicides showed a strong positive correlation. This trend was independent of the (C) region of origin or (D) the taxonomic identification of each isolate.

To evaluate the overall impact of herbicides across isolates and sampling locations, we determined the MS for each isolate and herbicide combination (see Methods for details). Most isolates had their MS values at 30 or 45 h of exposure, especially for glyphosate at the maximum concentration tested (Table 2). Figure 4 shows MS values of isolates grouped by their sampling region: Candelária, Alegrete and Quarta Colônia (which is subdivided into Nova Palma and Faxinal do Soturno subregions). Nova Palma and Alegrete presented the highest MS averages for both herbicides. For glyphosate, a *one‐way* ANOVA indicated significant MS differences among regions (*F* = 4.451, *p* = 0.0098), and the Tukey post hoc test indicated that Nova Palma had a significantly larger MS value than Candelária, Faxinal do Soturno, and the reference strains (Figure 4A). For 2,4‐D treatment responses, we also detected a significant difference among groups (one‐way ANOVA, *F* = 3.664, *p* = 0.021), with Nova Palma having higher MS values than the reference strains (Figure 4B). This variation among collection regions could suggest that the agricultural activity in each site has impacted the tolerance profiles of GAS bacteria.

**TABLE 2:** Maximum survival (MS, log2 scale) values (along with MS time and concentration) extracted from the survival curves to glyphosate and 2,4‐D of all Guarani Aquifer System isolates and reference strains.

Identification	Isolate	Glyphosate			2,4‐D		
		MS	MS time (hours)	MS concentration (mM)	MS	MS time (hours)	MS concentration (mM)
*Lysinibacillus*	C15	2.891	20	8	2.539	45	1.2
	GRU33	0.378	5	4	0.566	5	1.5
	MEL33	1.444	30	6	2.475	30	1.2
	RF3121	4.472	45	6	4.300	45	1.8
	VB1	1.816	45	8	1.438	20	1.2
*Bacillus*	CAV19	3.700	45	8	5.760	30	1.2
	CAV23	1.000	30	4	0.941	30	1.8
	C14	0.151	30	6	−0.364	30	1.2
	VB5	1.084	45	8	1.170	20	1.8
*Pseudomonas*	CAP2	2.718	45	6	3.472	45	1.2
	C9	2.660	20	4	1.687	45	1.8
	VB4	1.029	30	8	1.104	45	1.2
*Enterococcus*	CAP5	0.0566	20/30	8	−0.531	5	1.2
*Staphylococcus*	CAE1	3.459	20	8	3.711	20	1.2
*Leuconostoc*	CAV211	3.523	20	8	3.181	30	1.2
Unidentified	CAP3	4.419	45	4	1.740	45	1.2
	CAP6	1.782	45	8	0.433	30	1.2
	CAP7	1.795	45	8	1.753	45	1.8
	CAP10	3.536	45	8	1.787	20	1.5
	CAV28	4.186	20	8	2.834	30	1.8
Unsupported *Lysinibacillus*	C11	0.774	30	8	0.816	30	1.2/1.8
	RF311	5.514	45	4	3.472	30	1.8
	MEL13	10.541	45	8	11.522	45	1.2
*Bacillus cereus* ATCC 33019		1.091	5	4	−0.630	30	1.8
*Pseudomonas aeruginosa* ATCC 27853		2.524	45	4	0.526	45	1.8
*Enterococcus faecalis* ATCC 29212		0.014	5	4	−0.022	5	1.5

Since individual isolates seemed to show a similar tolerance trend to both herbicides—for example, an isolate that was promoted by glyphosate tended to also exhibit growth promotion to 2,4‐D—we examined whether this correlation persisted across isolates (Figure 4C,D). For this, we performed a linear regression on MS data from each of the 23 GAS isolates and the 3 reference strains, comparing MS in glyphosate and 2,4‐D treatments while grouping isolates by region of origin (Figure 4C) or genera (Figure 4D). We found a strong positive correlation between the MS values of both herbicides (Pearson correlation test, *t* = 10.078, *p* < 0.001, correlation coefficient = 0.899), with a linear regression slope approaching 1 (*R* ^2^ = 0.801, slope = 0.998, *p* < 0.001). These data indicate that glyphosate and 2,4‐D elicit a similar response on growth trends of individual isolates, regardless of their region of origin or taxonomic identification.

### 3.3 Antimicrobial Susceptibility

To investigate whether herbicide tolerance could correlate with survival to other antimicrobial agents, we first investigated the antibiotic susceptibility profiles of GAS isolates. For this, we used the disk‐diffusion assay to screen taxonomically identified isolates (*Lysinibacillus*, *Bacillus*, *Pseudomonas* and *Enterococcus*), as summarised in Table 3. The isolates identified as *Staphylococcus* and *Leuconostoc* were not included in these tests. *Staphylococcus* is described as strongly related to human microbiota (Byrd et al. 2018) or infections (Argemi et al. 2019) and rarely associated with microbial communities from aquatic environments. *Leuconostoc* is a genus native to plants, and it can also be found in silage and fermented food products (Poulsen et al. 2020), also described as potentially pathogenic to humans (Ino et al. 2016), without reports of occurring frequently in aquatic environments. The five unidentified bacteria and the three ‘unsupported *Lysinibacillus*’ isolates were also excluded from this analysis, as it demands a reliable taxonomic identification to be performed.

**TABLE 3:** Antimicrobial susceptibility profiles of the 13 taxonomically identified isolates, along with reference strains.

Identification	Isolate	Resistance profile (disk‐diffusion assay)	MIC (μg/mL)							MAR index
			MER	CAZ	ERY	TOB	CLI	ATM	VAN	
*Lysinibacillus*	C15	ERY, LZD, IPM, VAN, CLI	—	—	2	—	4	—	8	0.625
	GRU33	—	—	—	—	—	—	—	—	—
	MEL33	—	—	—	—	—	—	—	—	—
	RF3121	MER, ERY, LZD, IPM, VAN	8	—	8	—	—	—	4	0.625
	VB1	IPM, CLI	—	—	—	—	4	—	4	0.25
*Bacillus*	CAV19	—	—	—	—	—	—	—	—	—
	CAV23	—	—	—	—	—	—	—	—	—
	C14	MER, VAN	16	—	—	—	—	—	8	0.25
	VB5	IPM	—	—	—	—	—	—	—	—
*Pseudomonas*	CAP2	ATM	—	—	—	—	—	32	—	—
	C9	ATM, CAZ, FEP, TOB, LVX	—	8	—	2	—	64	—	0.45
	VB4	AMK, ATM, CAZ, FEP, TOB, GEN	—	8	—	2	—	32	—	0.54
*Enterococcus*	CAP5	—	—	—	—	—	—	—	—	—
*Bacillus cereus* ATCC 33019		—	—	—	—	—	—	—	—	—
*Pseudomonas aeruginosa* ATCC 27853		—	—	—	—	—	—	—	—	—
*Enterococcus faecalis* ATCC 29212		—	—	—	—	—	—	—	—	—

*Note:* For the disk‐diffusion test, the antibiotics to which isolates were resistant are indicated (Resistance profile). For isolates that showed resistance in this initial assay, the minimum inhibitory concentration (MIC) of seven antibiotics is indicated. The multiple antibiotic resistance (MAR) index is indicated for isolates that were resistant to at least two antibiotics.

Abbreviations: —, sensitive; AMK, amikacin; ATM, aztreonam; CAZ, ceftazidime; CLI, clindamycin; ERY, erythromycin; FEP, cefepime; GEN, gentamicin; IPM, imipenem; LVX, levofloxacin; LZD, linezolid; MER, meropenem; TOB, tobramycin; VAN, vancomycin.

**FIGURE 5:**
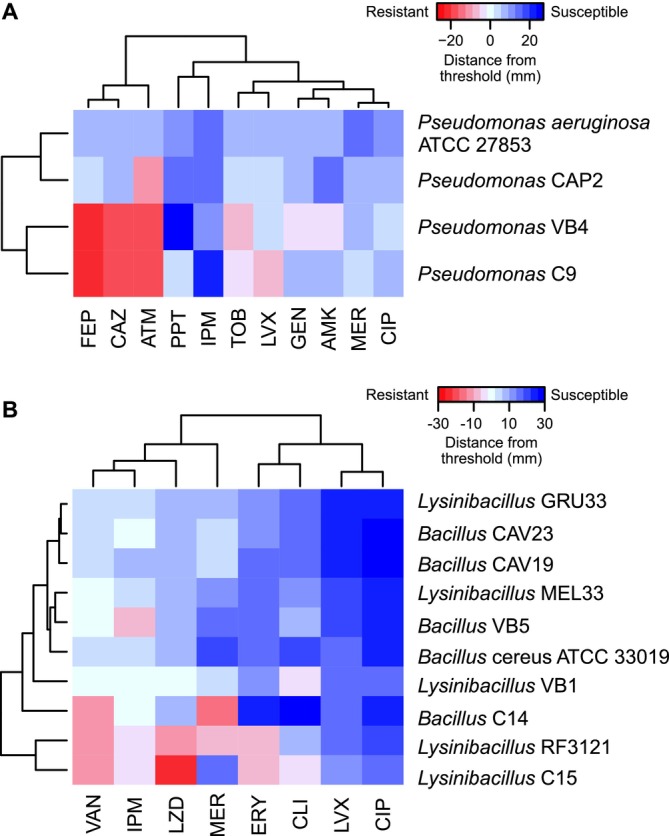
Antibiotic resistance profiles according to disk‐diffusion assays. (A) Isolates from *Pseudomonas* genus and (B) isolates from *Bacillaceae* family. The heatmaps represent the difference between the observed inhibition halo and the resistance threshold for each antibiotic/strain combination, such that positive (blue) and negative (red) values indicate susceptibility and resistance, respectively.

To illustrate the results from the disk‐diffusion assay for *Pseudomonas* and *Bacillaceae* isolates, we created heatmaps based on the diameters of inhibition zones (Figure 5). The colour scale represents the distance of the inhibition halo and the resistance threshold, such that positive values indicate sensitivity and negative values indicate resistance. All isolates identified as *Pseudomonas* were resistant to at least one of the 11 tested antimicrobials. Isolates C9 and VB4 were resistant to 5 and 6 drugs, respectively, which were mainly aminoglycosides and beta‐lactams (Table 3, Figure 5A). *Pseudomonas* was the genus that presented resistance to the highest number of drugs (a total of seven antibiotics for the three isolates), with a MAR index of 0.45 for C9 and 0.54 for VB4 (see Methods for details). Resistance to ceftazidime and tobramycin (MIC values of 8 and 2 μg/mL) was confirmed for both C9 and VB4 isolates. For aztreonam, MIC values were 64 μg/mL for C9 and 32 μg/mL for the other two isolates, whereas the resistance threshold for *Pseudomonas* spp. is set to 16 μg/mL according to the EUCAST Clinical Breakpoint standard.

Isolates from the *Bacillaceae* family showed a heterogeneous response to antibiotics (Figure 5B). Although the number of drugs tested for *Bacillaceae* (a total of 8) was lower compared to *Pseudomonas* isolates, the MAR index of both *Lysinibacillus* C15 and RF3121 was the highest among all resistant isolates, reaching 0.625 (Table 3). Interestingly, isolate C15 exhibited resistance to five antibiotics of different classes and distinct intracellular targets. VB1 was resistant to 2 antibiotics (MAR index = 0.25) and the other 2 *Lysinibacillus* isolates were susceptible to all drugs tested. Regarding *Bacillus* isolates, VB5 was resistant to only one antimicrobial (imipenem), C14 to meropenem and vancomycin (MAR index = 0.25), and the other two isolates were sensitive to all drugs tested.

We also evaluated the antimicrobial susceptibility of the *Enterococcus* isolate CAP5, which was sensitive to all 6 drugs tested. Moreover, all reference strains ( *B. cereus* ATCC 33019, *E. faecalis* ATCC 29212 and *P. aeruginosa* ATCC 27853) were sensitive to all antibiotics tested (Table 3, Figure 5). Therefore, although resistance profiles varied among strains, several GAS bacterial isolates showed resistance to several antibiotic drugs.

### 3.4 Tolerance to Herbicides and Resistance to Antimicrobials

**FIGURE 6:**
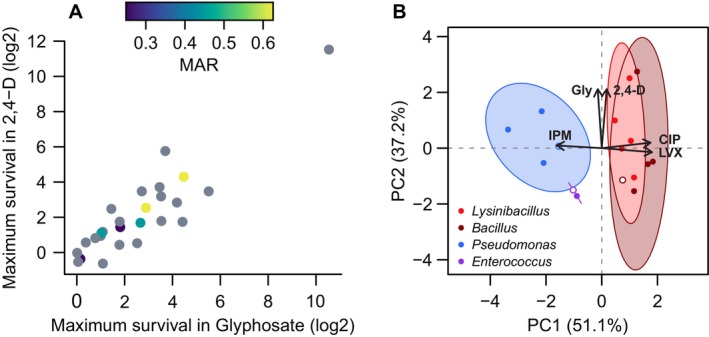
Susceptibility to herbicides and antibiotics across GAS isolates. (A) Visualisation of the linear regression presented on Figure 4C,D combined with the multiple antibiotic resistance (MAR) index as a colour scale. Each point represents a bacterial isolate (23 from the GAS and 3 reference strains). Isolates in grey did not have MAR index calculated (were sensitive to all antibiotics, resistant to only one drug or could not be tested via disk‐diffusion assay due to the lack of taxonomical identification). Data normalisation, linear regression and Pearson correlation information are the same presented for Figure 5. (B) Principal component analysis (PCA) distances among isolates according to MS and antibiotic sensitivity. The analysis comprised 5 axes: MS values for glyphosate and 2,4‐D, as well as diameters of inhibition zones for the broad‐spectrum antibiotics imipenem (IPM), ciprofloxacin (CIP) and levofloxacin (LVX). Blank circles indicate the reference strains for each group. The ellipses were calculated with 95% confidence intervals (PERMANOVA test, *p* < 0.05); the *Enterococcus* ellipse is a straight line, as there are only 2 isolates of this genus. The arrows represent the direction of each of the 5 axes of this analysis.

The linear regression between glyphosate and 2,4‐D MS values (Figure 4C,D) was used to include the MAR index of isolates that were resistant to at least 2 antimicrobials (Figure 6A). It was possible to observe that the 2 isolates with the highest MAR indices presented high MS values for both herbicides, whereas those with minor MAR indices had low MS values. This suggests a possible co‐occurrence of tolerance to herbicides and resistance to antibiotics, at least for some isolates, which merits further investigation.

To verify a possible relationship between tolerance to herbicides and resistance to antimicrobials among the GAS isolates (and reference strains), we performed a principal component analysis (PCA) on MS values from herbicide survival experiments along with the diameter of the inhibition zones from disk‐diffusion assays (Figure 6B). Five axes were included in this analysis: MS values for the two herbicides and inhibition to broad‐spectrum antibiotics imipenem (IPM), ciprofloxacin (CIP) and levofloxacin (LVX). The results from this analysis explained 88.3% of the total variance in our data. The first principal component (PC1) explained 51.1% of the variance, producing a separation among isolates that coincided with their taxonomical identification, as the *Bacillaceae* genera formed a distinct group from a *Pseudomonas* cluster and from *Enterococcus* isolates. The second principal component (PC2) explained 37.2% of the variance, mostly indicating the variability within these groups.

The arrows in Figure 6B represent the direction of each of the 5 axes in the PCA. These arrows are mostly aligned with the *x* and *y* axes, indicating that PC1 may explain the variance promoted by differences in antibiotic resistance, whereas PC2 represents the variance among isolates regarding tolerance to herbicides. They also indicate that IPM resistance data is opposed to that from CIP and LVX. It was possible to observe that antibiotic resistance was clearly distinct between the *Bacillaceae* cluster and the other isolates, whereas herbicide tolerance did not show a particular trend across taxa.

Regarding a possible cross‐tolerance pattern between herbicides and antibiotics, the *Pseudomonas* isolates that were resistant to multiple antibiotics also showed herbicide tolerance (VB4 and C9). The same was observed for *Lysinibacillus* C15, a herbicide‐tolerant isolate that showed resistance to five antibiotics, and the herbicide‐promoted *Lysinibacillus* RF3121, that showed equal levels of drug resistance. However, this relationship was not clear across all *Bacillaceae* isolates, since they exhibited a wide variance in their response to glyphosate and 2,4‐D (Figure 6B). *Bacillus* CAV19, for example, was susceptible to all tested antibiotics but promoted by herbicides (Figures 2 and 5). Taken together, these results suggest that herbicide tolerance is common among GAS isolates and often accompanied by antibiotic resistance, although cross‐tolerance patterns might be affected by taxon‐specific properties.

## 4 Discussion

The GAS represents one of the main underground reservoirs in South America, supplying freshwater for agricultural and industrial activities as well as household consumption (Sindico et al. 2018). However, there is evidence that the widespread use of herbicides on farmlands above might have led to the contamination of this system (Chiarello et al. 2017). To investigate the impact of such possible contamination on microbial communities, we evaluated the susceptibility of bacterial isolates from the GAS to herbicides. Our findings demonstrate that herbicide tolerance—and even growth promotion due to herbicide exposure—are commonly found among these isolates, and in some cases can co‐occur with resistance to multiple antibiotic drugs.

Out of the 23 isolates we investigated, 15 could be taxonomically identified into 6 different genera, with most being classified as *Lysinibacillus* (Schönbrunn et al. 2001), *Bacillus* (Talha‐Mar Soluções Ambientais 2010) and *Pseudomonas* (ANVISA 2018). Species from these genera are described as widespread, occurring very frequently in aquatic environments (Vilas‐Boas et al. 2024; Souza et al. 2023), and easily culturable from different sources. Several species of *Enterococcus* have also been frequently found in natural environments (Byappanahalli et al. 2012). The systematic review on Aquatic Microbiota by Souza et al. (2023) indicated *Pseudomonas*, *Bacillus* and *Enterococcus* among the main microbial genera reported in impacted freshwater environments (Souza et al. 2023). Moreover, *Bacillus* and *Lysinibacillus* species present the ability to form spores, which occurs as a natural defence of the bacteria to disturbances and in diverse ecological niches (Alcaraz et al. 2010; Gauvry et al. 2017). Considering the widespread and abundant occurrence of bacterial genera isolated in this study in aquatic environments, their phenotypic traits of tolerance to herbicides and resistance to antimicrobials represent a finding of great concern in terms of environmental, animal and human health. Furthermore, *Staphylococcus* and *Leuconostoc* isolates also appeared in our analysis. The genus *Staphylococcus* is commonly associated with mucous membranes, as a skin commensal (Byrd et al. 2018) or as a human pathogen (Argemi et al. 2019). *Leuconostoc* is usually found in plants, fermented food (Poulsen et al. 2020), and eventually as an opportunistic pathogen (Ino et al. 2016).

The high percentage of herbicide‐tolerant and herbicide‐promoted isolates indicates that most of these bacteria may have metabolic pathways that enable them to cope with the presence of glyphosate and/or 2,4‐D in their environment. Thus, these results indicate that at least part of the bacteria from the GAS may present such features, which can be positively selected by the presence of these compounds in the environment. However, some isolates presented a significant decrease in survival when exposed to these agrochemicals, including *B. cereus* and *Enterococcus* reference strains, which additionally showed large differences in survival over time. Moreover, most *Bacillus* isolates from GAS were susceptible to the herbicides, whereas CAV19 was promoted by both chemicals, indicating that for this bacterial genus this may be a strain‐specific trait. Additionally, as far as we could screen, the literature does not present any information that could explain *Bacillus* susceptibility to 2,4‐D or glyphosate. We only found data on the significant susceptibility of different *Bacillus* strains to other herbicides, like Imazapyr and Imazethapyr and Mesotrione. For Imazapyr and Imazethapyr, the authors indicate that *Bacillus* strains may present the target enzyme more sensitive to the action of these herbicides compared to other genera tested (Forlani et al. 1995). For Mesotrione, Dobrzansky et al. (2018) reported that the susceptibility of *Bacillus* strains was related to high oxidative stress (Dobrzanski et al. 2018), which can also occur due to other stressors, like glyphosate and 2,4‐D. Regardless of the underlying causes, both tolerance (which may even lead to increased survival) and decreased survival are considered important issues regarding the structure of microbial communities in aquatic environments, as previously discussed (Jørgensen et al. 2018). Additionally, we observed a strong positive correlation between MS in glyphosate and 2,4‐D, suggesting that tolerance to one herbicide might coincide with survival to other similar compounds.

Similar findings have been reported on the ability of bacteria to not only tolerate the presence of pesticides, but also to use them as a nutrient source. *Pantoea ananatis* , isolated from agricultural soil, resisted and grew in the presence of the selective herbicide mesotrione (Prione et al. 2016). Glyphosate‐based herbicides and their metabolites are degraded in different environmental matrices in contaminated niches via bacterial enrichment approaches (Singh, Kumar, Gill, et al. 2020). 2.4‐D mitigation strategies also rely on bacterial metabolic pathways to use and degrade this compound (Kumar et al. 2016). Moreover, *Pseudomonas* spp. were reported as presenting the ability to use glyphosate as a nutrient source for their growth (Singh, Kumar, Datta, et al. 2020) and *Lysinibacillus* has also been described as capable of biodegrading different herbicides (Reyes‐Cervantes et al. 2021). Our data indicated that one *Pseudomonas* isolate (CAP2), from the Alegrete region, was tolerant to both glyphosate and 2,4‐D, with some level of growth promotion. The other two *Pseudomonas* isolates (both from the Candelária region) were also tolerant to these herbicides. While it is possible that the sampling region may have some influence on these differences, underlying metabolic differences between isolates may play a role, since each of them showed a close phylogenetic relationship with a distinct species of *Pseudomonas*.

MEL13 (unsupported *Lysinibacillus*) presented the largest growth promotion among all isolates, showing a concentration‐ and time‐dependent response to both herbicide treatments. Importantly, this isolate was obtained from the sampling site MEL (Quarta Colônia region), in which 2,4‐D has been previously detected in concentrations up to 0.004 μg/mL (Soares 2019). Although this concentration is much lower than we tested in vitro, it is possible that the presence of 2,4‐D in sub‐lethal doses may have promoted the emergence of tolerance to this herbicide, and also to other stressors (like antibiotics), among GAS bacteria (de Castro Marcato et al. 2017). Whereas no data on glyphosate detection is available in literature for all sampling sites, our data indicated that, considering each isolate, the level of tolerance or sensitivity for both herbicides tends to be similar (Figure 4C,D). These results indicate that at least some isolates tested may be adapted to tolerate glyphosate and/or 2,4‐D, pointing to ecological concern regarding the structure of aquatic microbial ecosystems as well as human health (Jørgensen et al. 2018). In this sense, it is important to note that MS values from all isolates differed significantly by their region of origin, especially for Nova Palma isolates under glyphosate treatment. The Nova Palma region presents an intense agricultural activity for soybean production, on which both glyphosate and 2,4‐D are the most used agrochemicals (Feix et al. 2022). This could explain this finding, at least in part, since Candelária and Faxinal do Soturno also present soybean crops, but at lesser extension, whereas Alegrete presents mainly cattle production (Feix et al. 2022). However, the large heterogeneity that we observed among isolates, even those from the same region, suggests that the microbial response to these herbicides may be influenced by a combination of environmental factors and species‐ or even strain‐specific traits (Kurenbach et al. 2018; Shahid and Khan 2022).

No objective criteria have been established to classify bacteria as tolerant or resistant to herbicides, and these terms are often (but not always) used as synonyms. Nevertheless, some authors made suggestions regarding this issue. Bellinaso et al. (2003) proposed that ‘tolerance and resistance against pesticides in general is attributed due to physiological changes that induce microbial metabolism to follow a new metabolic pathway that help organisms to bypass a biochemical reaction, which could otherwise be inhibited by some specific pesticides’. Additionally, resistance could be due to the emergence of mutations inherited by different strains of microbes (Herman et al. 2005). Curutiu et al. (2017) propose that the occurrence of tolerance or resistance to herbicides among bacteria is perhaps a unique feature, which is regulated both genetically and physiologically. Some studies also suggest that microbial strains developing resistance to pesticides are capable of frequently degrading them (Herman et al. 2005; Shahid and Khan 2017), or use ‘resistance’ to refer to the ability of bacteria to grow in the presence of herbicides, irrespective treatment duration (Pileggi et al. 2020). Some of these criteria are used to define bacteria as resistant, strongly tolerant, highly tolerant or hyper tolerant to herbicides, whereas others consider bacterial tolerance to the maximum tested concentration of herbicides to define them as resistant to these chemicals (Jørgensen et al. 2018). As our survival tests were performed in 0.9% saline, and thus did not contain any nutritional source except the tested herbicides, the isolates that presented significantly increased survival (like MEL13), might have used these herbicides as a sole nutrient/carbon/energy source, through a biodegradation process. Thus, based on the criteria of ‘growing’ ability, we could define these isolates as herbicide‐promoted (or even resistant) when exposed to the tested herbicides. Moreover, this ability revealed a biotechnological potential for such bacteria, which must be further tested for their application in bioremediation processes adapted to aquatic environments. Our data also demonstrated that many isolates exhibited growth promotion at the maximum tested concentrations of the herbicides, which could be another indicative of the use of these molecules as a source of nutrients.

Herbicide application has been reported to potentially contribute to antibiotic resistance (Liao et al. 2021). The exposure of *E. coli* and *S. typhimurium* to commercial herbicides of different classes promoted a decrease in their susceptibility to antimicrobials (Brigitta et al. 2015). We found antibiotic resistance among many of our isolates. When we included the MAR indices in the regression analysis of both herbicide MS values, it was possible to observe that the isolates with the highest MAR indices presented high MS values, while those with the lowest MAR values were among the isolates with mild to low MS values, indicating that a relationship between tolerance to herbicides and resistance to antibiotics was detected for some isolates.

Of the isolates that exhibited resistance to antibiotics, many had MIC values far above the clinical threshold for resistance. *Pseudomonas* was the genus that showed resistance to the highest number of antibiotics tested. Nevertheless, *Lysinibacillus* isolates presented the highest MAR indices (0.625), which is also an important parameter to be considered. A common occurrence of antibiotic resistance was not expected for isolates from such a large aquatic environment. However, as the collection sites were located in areas of intense agricultural and livestock activity, these may be a source of chemicals inducing changes in the susceptibility profile of native bacterial strains (Berendonk et al. 2015). Even for antibiotics occurring in low concentrations in terrestrial and aquatic environments (Chow et al. 2021), it is already reported that the spread of faecal material (via sewage effluents and animal waste) has contributed to the contamination of most ecosystems around the planet with antibiotic‐resistant bacteria and resistance genes (Chow et al. 2021; Karkman et al. 2019; Williams et al. 2022). Furthermore, the spread of antibiotic resistance genes in the environment can promote an increment in the resistome content (Chen et al. 2021). Groundwater reservoirs are susceptible to this, since they maintain permanent connections with surface water and terrestrial environments (Pulido‐velazquez et al. 2007). Significant changes in groundwater microbial communities were reported due to river water infiltration due to a flood event, in which environmental conditions had a larger impact on this microbial community than dispersal of taxa from the river into the aquifer (Fillinger et al. 2021). So, considering the context of our study, it is plausible to infer that the herbicide‐tolerant and antibiotic‐resistant bacteria may originate mainly from surface‐polluted environments. Nevertheless, considering the historical wide use of herbicides and antibiotics in crops and livestock production during (at least) the last five decades in this Brazilian region (Feix et al. 2022), as well as the data from previous studies on antibiotic‐resistant bacteria and genes in groundwater from different locations (Zainab et al. 2020; Andrade et al. 2020; Anand et al. 2021; Junaid et al. 2022), it is also possible that some tolerant/resistant strains may be selected in the aquifer environment.

Low susceptibility to antimicrobials has been observed in *P. aeruginosa* from environmental origin (Laborda et al. 2021). Conversely, studies with environmental and clinical isolates of *P. aeruginosa* found that the environmental bacteria were significantly more susceptible to antibiotics than those of clinical origin (Gholami et al. 2017; Ramsay et al. 2019). Nevertheless, the environment plays a crucial role in both evolution and transmission of resistance (Reyes‐Cervantes et al. 2021), and it is not possible to predict where and under what circumstances the critical steps for antibiotic resistance will occur and what new forms of resistance will appear (Larsson and Flach 2021). Moreover, all *Pseudomonas* isolates in our study showed to be resistant to aztreonam (the only antibiotic to which an entire clade of bacteria showed resistance). Resistance to aztreonam by environmental *Pseudomonas* strains was reported by Luczkiewicz et al. (2015), in which most isolates were from wastewater and the marine coastal zone.

Moreover, our PCA suggested a phylogenetic‐related response to antibiotics, independently of the region of origin of the isolates. This taxon‐related pattern was not observed in the response to herbicides. As previously discussed, it has already been reported that at least *Pseudomonas* and *Lysinibacillus* can tolerate and even use herbicides as a nutrient source (Singh, Kumar, Datta, et al. 2020; Reyes‐Cervantes et al. 2021; Gaur et al. 2019). Studies using different taxonomic groups against a single or set of herbicides along with the susceptibility profile test to antimicrobials are still scarce. Our data also indicated that, even without a general relationship between tolerance to herbicides and resistance to antibiotics among the isolates, the two main taxonomic groups analysed presented at least one isolate with both properties. This result reinforces that such combined metabolic feature occurs within the microbial communities of GAS water. Moreover, the combined tolerance to herbicides and antibiotic resistance did not seem to depend on region/collection site. However, it does not indicate that environmental factors may have an irrelevant importance on the bacteria's response to these chemicals. In fact, since our data revealed a pattern of tolerance to herbicides and resistance to antimicrobials for most tested isolates, we can hypothesise that this may be the pattern for at least some groups of bacteria in the microbial communities from different regions of the Guarani Aquifer. Thus, if herbicides‐tolerant bacteria from GAS can also be resistant to antibiotics, anthropogenic impacts in this aquifer may also extend to indirect selection of antimicrobial resistant bacteria, as already observed for surface environments (Kurenbach et al. 2018; Curutiu et al. 2017; Shahid and Khan 2022; Liao et al. 2021).

Most studies that evaluated the impact of herbicides on microorganisms, or the tolerance of microbial species to these agrochemicals, analysed bacterial isolates from soil, especially from rhizosphere microbiota (Shahid and Khan 2022; Liao et al. 2021). Even when species from aquatic environments were tested, the methods applied were biochemical (Gaur et al. 2019; Rovida AF da et al. 2021) or survival/growth curves based on optical densities (Dobrzanski et al. 2018; Rovida AF da et al. 2021). Other studies used the maximum concentration of herbicides that the isolates tolerated to evaluate them regarding this feature (Singh, Kumar, Gill, et al. 2020). To the best of our knowledge, our study is the first to raise data on the susceptibility to herbicides of bacterial isolates from an aquifer environment, employing survival measures based on CFU/mL counts. Moreover, recent studies used herbicides in sub‐lethal concentrations to verify if they changed antibiotics MIC values in these isolates (Brigitta et al. 2015; Kurenbach et al. 2017; Chow et al. 2021; Brigitta et al. 2015; Malagón‐Rojas et al. 2020). Differently from those, we analysed the bacterial response to herbicides and antibiotics independently, without any pre‐induction or selection, thus analysing in vitro the innate responses of the isolates to these biocides. Nevertheless, it is important to note that abiotic factors in their original environment may induce variabilities in the response we observed in our in vitro tests, as already reported in previous studies (Ramsay et al. 2019; Kurenbach et al. 2017). Shahid and Khan (2018) even propose that it may be impossible to generalise the elements that influence toxicity or tolerance of agrochemicals, not only in natural environments, but also along the different in vitro tests already performed by different studies.

Aquifers are large reservoirs of groundwater, considered one of the most important sources of safe freshwater for human consumption. Maintaining a community where microbes remain largely susceptible to herbicides and antibiotics can be beneficial for humans and ecosystems, as it helps control the spread of harmful organisms both locally and globally (Jørgensen et al. 2018). We found that most isolates from the GAS had tolerance or high tolerance to glyphosate and/2,4‐D, and resistance to at least one antimicrobial. Concerning the context of the One Health principle, there is a great need to manage susceptibility to antibiotics and pesticides as a valuable strategy for environmental sustainability, and human and non‐human animal health (Jørgensen et al. 2018; Humboldt‐Dachroeden and Mantovani 2021). In this context, our results raised relevant data, pointing to the importance of characterising microbes from unexplored environments as potential indicators of human environmental impact and/or remediators of contaminants.

## 5 Conclusions

This study presents the first characterisation of herbicide and antibiotic susceptibility of bacteria isolated from a deep aquifer, the South American Guarany Aquifer System. Herbicide tolerance to glyphosate and/or 2,4‐D commercial formulations was revealed to be high and common, since it was detected in 19 out of 23 bacterial isolates of genera *Bacillus*, *Lysinibacillu*s, *Pseudomonas* and *Enterococcus*. Among these, some also presented increased growth with the herbicides as sole nutrients, carbon and energy sources, indicating they may consume these compounds. Moreover, the tolerance to both herbicides presented significant correlation, as this tolerance was also correlated with antibiotic resistance of isolates. Considering the widespread occurrence of the bacterial genera studied in aquatic environments, their phenotypic traits of tolerance to herbicides and resistance to antimicrobials represent a finding of great concern in terms of environmental, animal and human health. The results suggest that herbicides may impact microbial communities' composition and functionality in aquifers, especially from regions of intensive agricultural activities. They also highlight the importance of studying the metabolic features of microbes from impacted environments as potential indicators of chemical contamination in these habitats, or as remediators of harmful xenobiotics, in line with the One Health principle.

## Author Contributions

**Carolina S. O. Silva:** data curation, investigation, formal analysis, writing – original draft, methodology, validation, visualization. **Sílvia D. Oliveira:** formal analysis, methodology, resources, supervision, validation, writing – review and editing. **Audrey M. Proenca:** formal analysis, data curation, validation, visualization, writing – review and editing, methodology. **Eduarda V. Abati:** methodology, data curation, investigation, writing – original draft, visualization. **Letícia Marconatto:** methodology, formal analysis, writing – review and editing, investigation. **Cássio S. Moura:** conceptualization, funding acquisition, resources, methodology, project administration, writing – review and editing. **Renata Medina‐Silva:** conceptualization, formal analysis, funding acquisition, project administration, resources, supervision, writing – review and editing, data curation, visualization.

## Conflicts of Interest

The authors declare no conflicts of interest.

## Supporting information


**Figure S1.** Map of South America region where the Guarani Aquifer System area expands, highlighting the Brazilian state Rio Grande do Sul (RS), in which this study was performed. The magnified area contains the three regions of water collecting sites, from which the bacteria tested in this study were isolated.
**Figure S2**. Relative survival to glyphosate and 2,4‐D, in semi‐log curves of isolates that presented no significant responses to the treatments, suggesting herbicide tolerance. (A) Taxonomically identified isolates; (B) unidentified or unsupported isolates. Error bars = mean ± relative error. Dashed line represents no change in growth, and shaded areas indicate the 95% confidence intervals.

## Data Availability

All data generated or analysed during this study are included in this published article and its supplementary information files. The 16S rRNA sequences from isolates that were taxonomically identified were deposited in the NCBI database: C9 (OM949960), C14 (OM949967), CAE1 (OM949968), CAP2 (OM949990), CAV19 (OM952179), CAV211 (OM952208), GRU33 (OM952259), MEL33 (OM952437), VB1 (OM952920) and VB4 (OM952921).
