# Supplementary material for: Tolerance to Herbicides and Resistance to Antibacterial Drugs of Bacterial Isolates From the Guarani Aquifer System (Brazil)
Source: Environ Microbiol. 2025 Jun 19;27(6):e70115. doi: 10.1111/1462-2920.70115 (PMC12178776; doi:10.1111/1462-2920.70115)
Supplement: Supplementary file 1 — Figure S1. Map of South America region where the Guarani Aquifer System area expands, highlighting the Brazilian state Rio Grande do Sul (RS), in which this study was performed. The magnified area contains the three regions of water collecting sites, from which the bacteria tested in this study were isolated. Figure S2. Relative survival to glyphosate and 2,4‐D, in semi‐log curves of isolates that presented no significant responses to the treatments, suggesting herbicide tolerance. (A) Taxonomically identified isolates; (B) unidentified or unsupported isolates. Error bars = mean ± relative error. Dashed line represents no change in growth, and shaded areas indicate the 95% confidence intervals. [file EMI-27-e70115-s001.docx]

**Supplementary material**

**
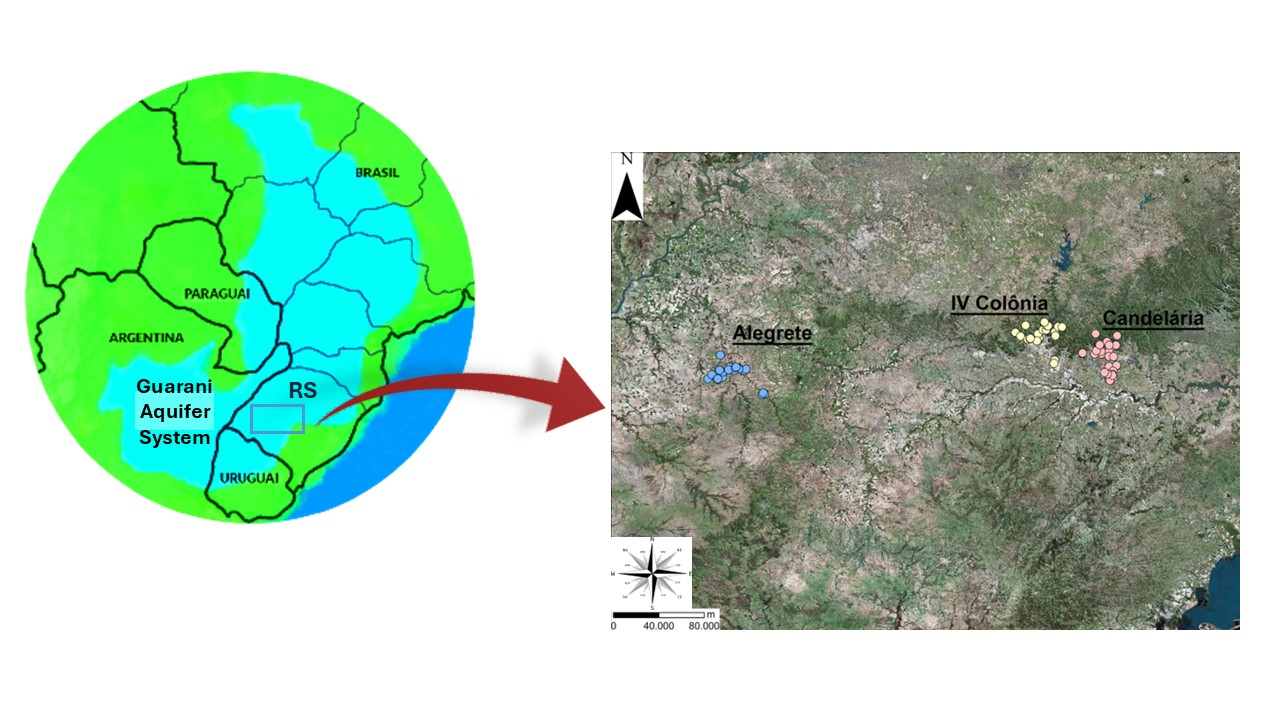
**

**Supplementary Figure 1** Map of South America region where the Guarani Aquifer System area expands, highlighting the Brazilian state Rio Grande do Sul (RS), in which this study was performed. The magnified area contains the three regions of water collecting sites, from which the bacteria tested in this study were isolated.


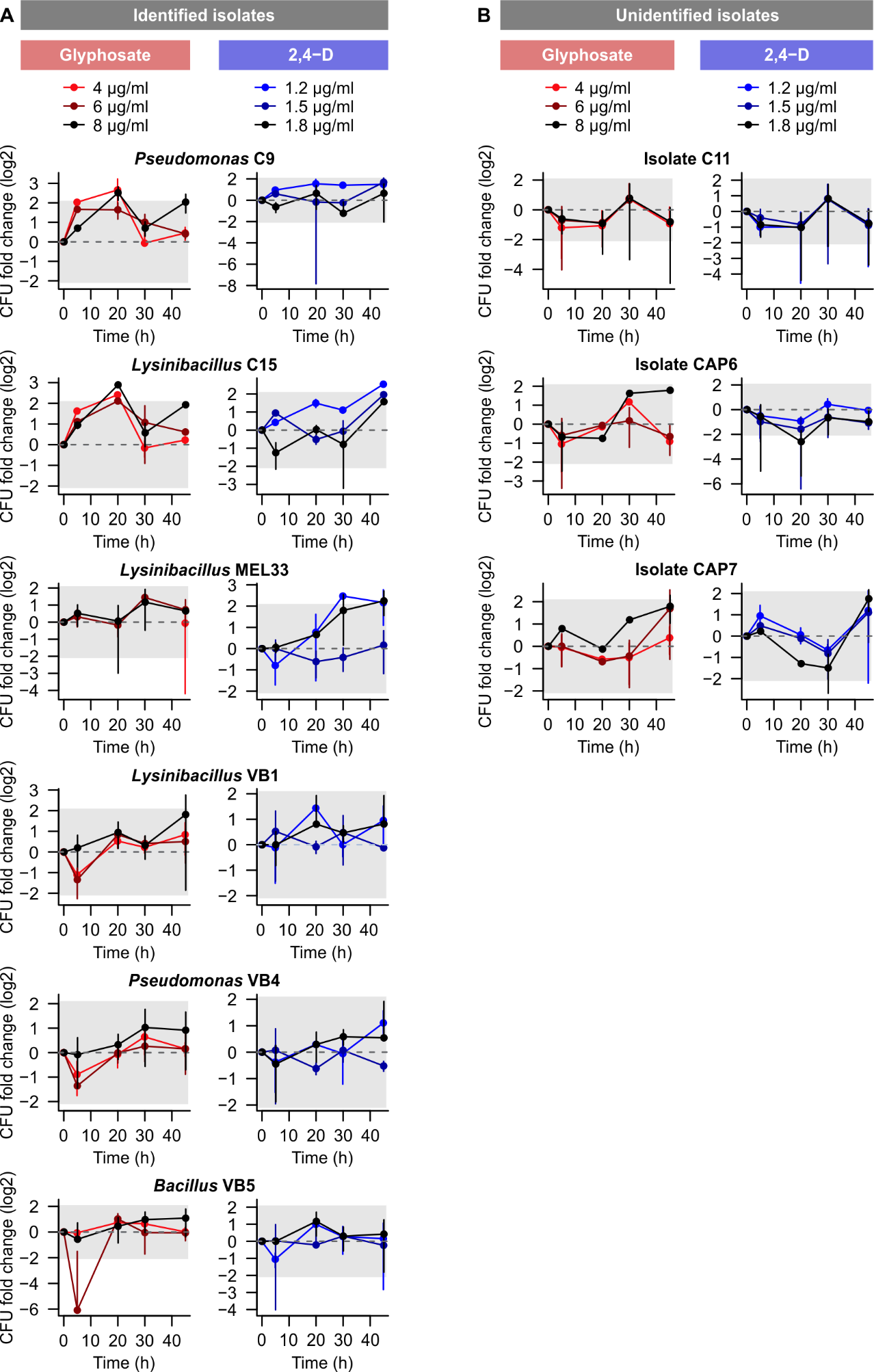


**Supplementary Figure 2** Relative survival to glyphosate and 2,4-D, in semi-log curves of isolates that presented no significant responses to the treatments, suggesting herbicide tolerance. (A) taxonomically identified isolates; (B) unidentified or unsupported isolates. Error bars = mean ± relative error. Dashed line represents no change in growth, and shaded areas indicate the 95% confidence intervals.
